# Impact of COVID-19 on renal replacement therapy: perspective from a Nigerian renal transplant centre

**DOI:** 10.11604/pamj.2022.42.90.33387

**Published:** 2022-06-02

**Authors:** Martin Chukwudum Igbokwe, Stephen Olabode Asaolu, Michael Obinna Muoka, Olalekan Olayinka Olatise

**Affiliations:** 1 Urology Unit, Department of Surgery, Zenith Medical and Kidney Centre, Abuja, Nigeria,; 2 Department of Clinical Research, Zenith Medical and Kidney Centre, Abuja, Nigeria,; 3 Nephrology Unit, Department of Medicine, Zenith Medical and Kidney Centre, Abuja, Nigeria

**Keywords:** COVID-19, renal replacement therapy, haemodialysis, kidney transplant, Nigeria

## Abstract

**Introduction:**

COVID-19 has had a huge impact on the health system and the world at large. Patients with kidney failure are a select group which have been affected significantly by the scourge of the disease. In the COVID-19 era, renal replacement therapy (RRT) in the form of dialysis and kidney transplantation required modifications in many centres in order to maintain high quality care and reduce infection rates among this susceptible group of patients. The objectives were to describe some of the challenges experienced in one of the leading renal care centres in Nigeria during the height of the COVID-19 pandemic and analyse the impact of practice changes on select outcomes.

**Methods:**

a retrospective cross-sectional review of haemodialysis activities and kidney transplantation among chronic kidney disease patients was done over a 15-month period ranging from April, 2019 to June, 2021. Data was extracted from the electronic media record (EMR) and analysed using SPSS version 22.

**Results:**

there was an initial significant drop in the number of haemodialysis sessions and kidney transplant surgeries by 16.7% and 66% respectively in the first 2 months of COVID-19 in our centre following the national lockdown. The mean monthly kidney transplant rate was 9±3.29 before the COVID-19 and the national lockdown, this figure reduced to 3.0±0.1 during the lockdown. Activities however normalized at 6 months following the initial lockdowns have remarkable exceeded pre-COVID numbers as at early 2021.

**Conclusion:**

after the initial drop in numbers of patients for haemodialysis and renal transplantation, there was an increase in numbers in the following months. It was instructive to put several steps in place in order to continue to offer high level RRT in the COVID-19 pandemic. RRT can safely be practiced in the COVID-19 pandemic.

## Introduction

The world is still coming to terms with the impact of the novel coronavirus 2019 (or coronavirus disease 2019 [COVID-19]). COVID-19 is caused by a newly identified enveloped RNA virus named SARS-CoV-2 [1]. This virus has proved to be highly virulent and is one of the most infectious agents known to mankind. Since its discovery in Wuhan, China in December 2019 there have been a total of 496 million cases of COVID-19 (in accordance with the applied case definitions and testing strategies in the affected countries) recorded, including 6.17 million deaths as of April 8^th^, 2022 [2]. This high mortality rate is compounded by the fact that majority of the fatal cases were found in the elderly, obese and individuals with significant systemic and respiratory co-morbidities. Nigeria has not been left out of the scourge of the disease, with 255, 468 confirmed cases, 249, 607 recovered cases and 3, 142 deaths as of April 8^th^, 2022 [3]. At the moment, every continent in the world has experienced cases of COVID-19 with varying incidence of recoveries and deaths.

COVID-19 has had an enormous impact on medical systems across the globe, with effects ranging from disruptions to complete shutdown of established medical models. In many countries, the overwhelming impact of COVID-19 on health care systems has resulted in limited resources like operating space, doctors, nurses and bed spaces for admissions from other medical conditions [4,5]. These resources were mobilized and re-deployed to care for the enormous population of COVID-19 patients in isolation centres and emergency units [6]. Also, the infection of health care providers due to inadequate protection apparatus has reduced the number available to care for patients [7]. This is especially significant in resource poor settings like Nigeria. In Nigeria, the healthcare system is further stretched by the mass exodus of healthcare professionals from the country in the years leading up to the pandemic [8,9]. Kidney transplant programs have suffered tremendously in the face of COVID-19 as have other transplant programs worldwide.

The field of renal care, especially dialysis and kidney transplant experienced disruptions, with organizations making necessary changes to protocols and policies in the wake of the pandemic. A major reason for this was the concern that pandemic conditions may not be optimal for dialysis and transplant patients considering the infectious nature of the virus, the invasive nature of the whole dialysis connection and the susceptibility of post-transplant patients to infections [10]. The presence of co-existing co-morbidities like diabetes mellitus is also a source of concern in this cohort of patients [11]. Additionally, studies appear to show that kidney transplant recipients are at a higher risk to contract COVID-19 than the rest of the population [11,12].

Despite these facts, some dialysis and kidney transplant programs have remained active in European countries where deceased donors make up a significant population of kidney donor pools [13]. In Nigeria, kidney transplantation has undergone a significant metamorphosis over the last two decades. Up from few tertiary hospitals who performed less than 50 kidney transplants per year, [14] presently there are multiple renal transplant centres with robust kidney transplant programs which have changed the outlook of kidney transplantation in Nigeria. These programs have taken a significant setback in the face of COVID-19. We do not yet fully understand the impact these practice changes have had on kidney transplant in Nigeria and the related effects on short term transplant outcomes. This article aims to describe some of the challenges experienced in one of the leading renal care centres in Nigeria during the height of the COVID-19 pandemic and analyse the impact of practice changes on select outcomes.

## Methods

**Study design, participants, and setting:** this study was carried out in Zenith Medical and Kidney Centre, Abuja, Nigeria. A retrospective review of haemodialysis activities and kidney transplantation among chronic kidney disease patients was done over a 15-month period ranging from the pre-COVID-19 era (April, 2019) to June, 2021.

**Variables, data sources and bias:** data were collected from electronic medical records, including monthly reports of dialysis and transplant activity. All data collected were included in the analysis. This data allowed for comparison in number of haemodialysis and kidney transplant activities during the lockdown period and afterwards. Other peculiarities during the study period on the haemodialysis and transplant activities were also described in this paper.

**Statistical methods:** data were entered into and analysed using IBM SPSS Statistics for Windows, Version 20.0. (Armonk, New York: IBM Corp) and visualized using Microsoft Office Excel (Redmond, Washington: Microsoft Corp). Simple descriptive analysis was used to interpret the collected data. Some continuous variables were presented as mean ± standard deviation while comparative data for periods of time were presented in bar charts and trend lines.

**Ethical approval:** this study analysed secondary data from the electronic medical records of our transplant centre. The data were already anonymized in the output sheet therefore, no explicit consent to use this data was necessary. Ethical approval for the study was sought and obtained from the research and ethics review committee of the Federal Capital Territory Administration (FCTA) Department of Health with approval number FHREC/2021/01/159/14-12-21.

## Results

### Kidney transplant rates

**Figure 1:**
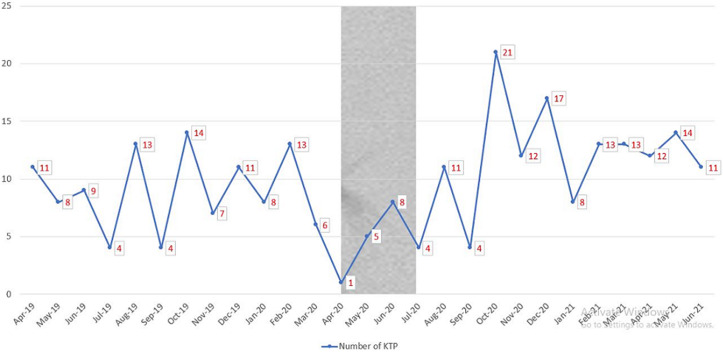
pattern of kidney transplant in the period under review (April 2019 till June 2021)

**Figure 2:**
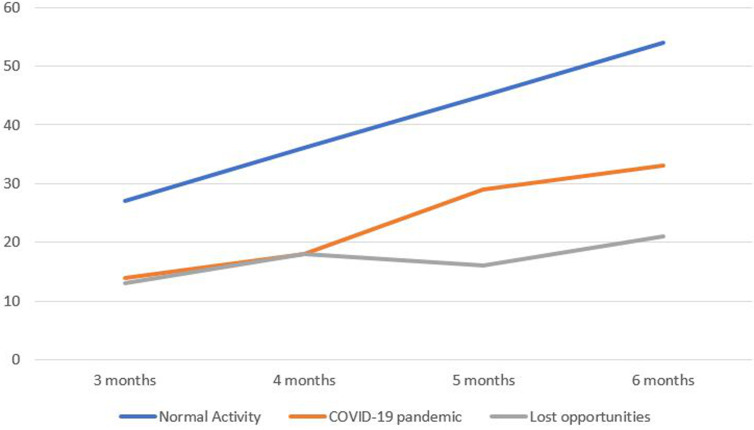
expected cumulative number of transplants performed in normal times and during the height of COVID-19 restrictions

For the duration of one year before the declaration of a lockdown in Nigeria (April 2019 till March 2020), there was a mean monthly living-related donor kidney transplants rate of 9±3.29. There was rapid decline in number of kidney transplantation surgeries performed during the initial 2 months of the COVID-19 lockdown in Nigeria with a transplant mean monthly rate of 3.0 ±0.1 (Figure 1). This indicates a significant loss in transplant opportunities. Figure 2 shows the number of expected kidney transplants based on projected activity in normal circumstances and during the height of COVID-19 restrictions, as well as the number of lost opportunities.

### Dialysis activity

**Figure 3:**
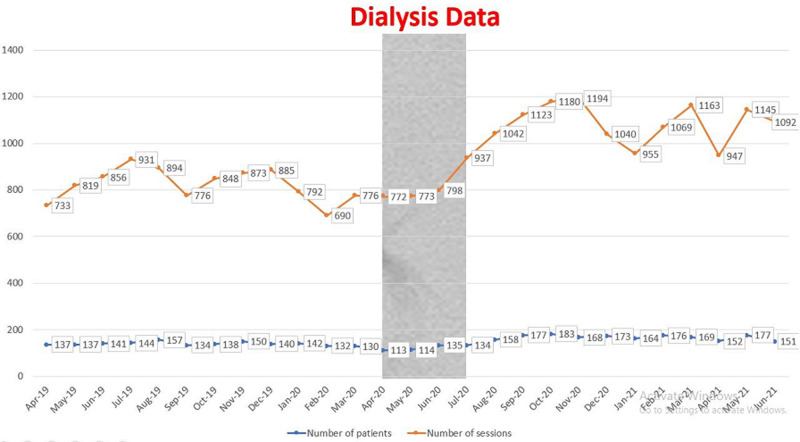
pattern of dialysis sessions in the period under review (April 2019 till June 2021)

It is expected that missed transplant opportunities would result in an increased number of patients on maintenance dialysis. Instead, in April and May 2020, there was a drop in the number of patients receiving haemodialysis, this number slightly increased in the next month (Figure 3). The average number of patients on dialysis was 135 from January 2020 to March 2020. However, this number dropped to 113 patients in the next couple of months.

## Discussion

### Impact of COVID-19 on the Kidney Transplant Program (Renal care Program)

This study has quantified the impact of the COVID-19 pandemic on renal care and transplant activity in a single centre in Nigeria.

### The End Stage Renal Disease (ESRD) patient on maintenance haemodialysis

Haemodialysis has been the mainstay of management for patients with end stage renal disease (ESRD) worldwide [15]. The last 3 decades have witnessed the birth of many more haemodialysis centres in Nigeria with the aim of meeting the dialysis requirement of the ever-rising number of chronic kidney disease (CKD) patients. Adequate haemodialysis for an ESRD patient by international guidelines is defined as three sessions per week, each session lasting from three to five hours [16,17]. In the face of COVID-19 and considering the vulnerable nature of the haemodialysis patient, various centres are expected to install measures to mitigate the spread, morbidity and mortality from COVID-19 among this special group of patients [18,19]. The pandemic affected the Nigerian CKD patient population negatively especially during the peak when the Nigerian government implemented a total lock down of almost all activities in the country. The total lock down limited people´s movement in an attempt to significantly reduce the spread of the disease with consequent adverse effect in the number of functional haemodialysis centres, access to the available facilities and having the means (financial) to afford dialysis services. Worldwide, the number of renal transplant patients diagnosed with COVID-19 was higher than in the general population [20]. Many factors make the ESRD patients on haemodialysis prone to contracting and dying from COVID-19. These include the fact that several individuals including staff and other patients may be using the same dialysis facility at the same time increasing exposure time for COVID-19 transmission. Also, the advanced age of most ESRD patients, presence of co-morbidities like diabetes, hypertension, a range of heart diseases and human immunodeficiency virus as well as an already immune-compromised state of health makes them at high risk to contract and perhaps die from COVID-19 [18,21]. Studies from China among haemodialysis patients actually showed a higher mortality rate (ranging from 13.3 to 16.2%) compared to the normal populace [22,23]. In the United States (US), a retrospective study of 7,948 patients on haemodialysis (out of which 438 (5.5%) were diagnosed of COVID-19), found that mortality was 24.9% in those with COVID-19 compared to 3.7% in those without the disease (P = <0.001). To this effect, it has become imperative to provide guiding frame-works with regards to haemodialysis in the COVID-19 era including educating ESRD patients and their health-workers, liberal screening of high-risk patients and their contacts, wide use of PPES and appropriate management of patients found positive for COVID-19 [24,25]. These difficulties for haemodialysis patients could be a cause of inadequate dialysis and a further hindrance to preparation for Kidney transplantation. Not much has been studied as regards COVID-19 and haemodialysis in Nigeria, but anecdotal experience from a single haemodialysis unit suggested that there was no change in the number of haemodialysis sessions per month however there was liberal testing of patients with fever, respiratory symptoms, and abnormal chest computerised tomography scan findings.

### Preparation for Kidney Transplant

The decision to continue the kidney transplant program was taken after careful consideration of donor risks in view of the absence of deceased donor program in Nigeria and the life changing nature of the procedure. The preparation and execution of kidney transplantation suffered some major setbacks during the height of the pandemic. Some of the encountered challenges include: the lockdown and embargo on inter-state travels imposed by the government of Nigeria limited many patients from gaining access to the hospital in order to run their hemograms, renal function tests, ABO compatibility tests and renal angiography. The lockdown affected the availability of consumables for other preliminary tests that can be done in the laboratories in country. Haemodialysis also became inadequate among many patients due to this movement restriction. Secondly, at that time there were no advanced molecular laboratories in Nigeria capable of running high quality human leukocyte antigen (HLA) testing, donor specific antibody titres and crossmatches, this meant that many transplant centres in Nigerian relied on foreign laboratories for credible results. Thirdly, kidney transplantation and haemodialysis require a strong haematological support in view of blood transfusion requirements with Zenith Medical and Kidney Centre, having the highest blood requirements in the whole Abuja metropolis. Blood drive campaigns and networking with other big haematological laboratories were significantly hampered during the lockdown period with only few voluntary donors able to make their way for blood.

These challenges demanded some strategy by the hospital management in order to sustain renal care for the large number of CKD patients requiring forms of renal replacement therapy during this period. Some of these modifications include: downsizing of the work force in the hospital to ease the implementation of social distancing. This was aimed to limit possible transmission of the disease. Apart from wearing personal protective equipment (PPEs), one of the recommendations by the US Department of Labour´s Occupational safety and health administration [26] in response to COVID-19 is that organizations should encourage their members of staff to work from home where they can, and that very high exposure risk workers like healthcare workers should be reduced to functional units (where possible) to further allow for workplace distancing. In a report by Ijarotimi *et al*. [27], doctors were encouraged to consult over the telephone for non-emergency cases and roasters were reorganized to limit the hospital contact of medical personnel.

Secondly, the hospital commenced a series of seminars and presentations (including making posters and short videos) about what is known about the virus and its transmission. There is a positive relationship between education and health. Health education and promotion is one of the frontline strategies effective against disease outbreaks [28-30]. It has been proven to contribute towards flattening of the epidemic curve in fast spreading and highly virulent pandemics. Lopes [31] reported in a webinar about how cities respond to COVID-19 that the pandemic curve flattened significantly following massive education campaign embarked upon by the government of South Korea.

Thirdly, PPEs were acquired and made available to the hospital staff on duty. Following guidelines by World Health Organization (WHO) [32], adequate and appropriate PPEs were sourced, acquired, and distributed to hospital staff on duty. An early cross-sectional study [33] of 420 healthcare professionals in Wuhan, China (the epicentre of the pandemic) concluded that PPEs offers appropriate effective protection for the study participants despite being at high risk of exposure, especially since there were no safe and effective vaccines available. An earlier study by Ran *et al*. [34] in the same location had posited that personal protective equipment to be worn for SARS-CoV-2 by healthcare workers should include protective masks, round caps, gloves, protective clothing, boot covers, and goggles or a face shield, the study found them effective in considerably reducing the risk of infection among local healthcare professionals.

Lastly, periodic screening for surgeons and theatre staff was commenced to allow for early detection and prompt isolation in case of infection with the virus. This is in line with extant guidelines by the government and also designed to further protect the patients from contracting the virus. Both patients and healthcare workers are at constant risk of nosocomial transmission of the COVID-19 virus, considering that the virus is airborne and very infectious [35]. The WHO [36] recommends syndromic surveillance and/or routine laboratory testing as a key strategy to prevent secondary transmission from health workers to patients, between health workers throughout health-care settings and from health workers to contacts outside of health facilities.

In our centre, there was a progressive decrease in transplant activities following the declaration and implementation of lockdown by the government as shown in Figure 1. The projected number of transplants did not recover immediately after the lockdown was lifted; this is despite the fact that there was a backlog of patients desirous of kidney transplant. However, there is an upward trend in transplant activity starting from September 2020, which has an obvious explanation. Due to the lockdown and travel restriction to countries that were choice destinations for Nigerians seeking kidney transplant, many patients have had to seek care from hospitals providing transplantation in Nigeria. European countries like the United Kingdom, France, Spain and Netherlands reported a significant decrease in transplant activity in the peak of the pandemic during the lockdown as a result of more than 50% decrease in organ donation [37-40]. In the United States (US), this figure dropped by more than 35% [41]. There is a noticeable trend of lower transplant activity in regions with higher infection rates, at some point some physicians in the US fully suspended live donation kidney transplant [42]. In South Africa [43], living donor kidney transplantation was in a standstill, resulting in a massive reduction in transplant activities as the deceased donor kidney transplantation forms a small portion of transplant programs in the country. This might be because South Africa has some of the highest rates of HIV and tuberculosis worldwide, there might be increased mortality if COVID-19 spreads in the population. However, the rates of haemodialysis sessions and kidney transplantation rose remarkably after the peak COVID-19 period and has been maintained as such till date.

**Limitations:** this is a single centre study in a super-specialized renal care centre in Nigeria, a multicentre study would have provided more robust data.

## Conclusion

Renal replacement therapy in the forms of haemodialysis and kidney transplantation provides a life-saving option for patients with ESRD and is increasingly available in Nigeria. The impact of COVID-19 was felt in the availability of these services in our centre during the study period which a rapid decline in number of haemodialysis sessions and kidney transplants done. These however peaked afterwards following re-strategizing and modified management protocols. While COVID-19 remains an international pandemic, it is important for RRT to continue in order to meet the needs of these highly vulnerable group of patients.

### What is known about this topic

- The COVID-19 pandemic significantly disrupted kidney transplantation in affected countries;
- Mortality among kidney transplant waitlisted patients is significantly increased during the height of the pandemic;
- Kidney transplant programs in Nigeria are still in early stages of development; centres offering kidney transplant are very few and there is no deceased donor program

### What this study adds

- This study quantified the impact of COVID-19 on the kidney transplant program in a Nigerian super-specialized renal care centre;
- Development of sustainable infrastructure and expertise should remain a priority for the kidney transplant program in Nigeria;
- This study shows the value of readily available healthcare data to inform strategic planning in the event of future crises.
